# Intussusception

**Published:** 1878-04

**Authors:** A. A. Hubbell

**Affiliations:** Leon, N. Y.


ART. II.—*A Case of Chronic Intussusception of Eleven Months Duration.* By A. A. HUBBELL, M. D., Leon, N. Y.

In the February No. of this Journal I presented the history of a case of acute intussusception with obstruction. Since writing that article, another case of the disease has terminated under my observation. It was one, however, of a very different character, being very chronic in its nature, and without obstruction. The symptoms were correspondingly different; and from their obscurity, no positive diagnosis could be agreed upon during life. The case did not, therefore, receive treatment suited to recovery; and it finally terminated in death. The report that I here give, then, is simply the *Natural History* of a case of chronic intussusception unmodified, except by the use of anodynes. I submit it as another *fact* in the study and history of the subject, showing the long duration to which the disease may extend, and the patient finally die from exhaustion, without obstruction, peritonitis, or any of the usual consequences ensuing.

Frank P———, aged 17 years, was, like the other case above referred to, convalescent from a mild attack of measles, when he began to complain of pain in his bowels on the 25th of February, 1877. His previous health had generally been excellent; and at this time he was able to be out of the house. Shortly before feeling the pain, he assisted in unloading some ice from a sleigh. The pain of which he complained was paroxysmal, lasting two or three minutes and then leaving him easy for ten to thirty minutes. His bowels moved quite regularly; his appetite was moderately good; and he was free from any disturbance of the secretions or excretions, as well as from feverishness or chilliness. Physical examination showed nothing abnormal, except a little fullness in the right hypochondrium. I will not take space to detail the treatment that I resorted to; but suffice it to say that it consisted mainly in tonics and anodynes, with an occasional laxative which always operated as desired. In fact, there was apparently no constipation, except what might be expected from the anodynes. The case continued with the above symptoms till the fifth week, the patient being about the house nearly all the time, when there appeared a well-defined tumor in the central part of the abdomen. It measured about two inches one way, and four, the other; its long diameter being transverse to the axis of the body. It was dull on percussion, somewhat tender on pressure, movable in all directions, and when lying over the aorta transmitted distinct pulsations. The patient was placed in bed, and the nurse applied warm fomentations to the abdomen. In a short time, to our surprise, the tumor disappeared. The next day the patient was allowed to get up, and the tumor again made its appearance in the region of the umbilicus, which disappeared as before, on his lying down. For two weeks the tumor would thus go and come, after which, it remained permanently near the umbilicus, whatever position of the body was assumed. It was observable that whenever the tumor was found in the central part of the abdomen, there was no fullness in the right hypochondrium, and vice versa; thus indicating the constant existence of the tumor, although, when it lay in the region of t hepatic flexure of the colon its outlines could not be clearly distinguished. Indeed, the fullness at this point seemed, at times, to be merely contraction of the upper part of the right rectus abdominis muscle.

After the second month, there began to arise some gastric disturbance, with loss of appetite, occasional nausea, and once in a few days, vomiting. At about the same time, the stools became diarrhœtic, only one or two occurring each twenty-four hours. The pain continued about the same, and required the daily use of anodynes. The patient's position was another characteristic symptom, namely, that of flexion of the thighs upon the body whether sitting or lying down. These symptoms continued without much change till November,the patient being about the house nearly every day, notwithstanding his increasing emaciation, and failure of strength. About this time his appetite almost entirely disappeared, the pain became more severe, the diarrhœa was increased having from three to ten passages in the twenty-four hours, and he complained bitterly of sore mouth, although nothing but a general redness could be seen. He was occasionally nauseous and every few days would vomit; getting only temporary relief from opiates, the more severe symptoms continued, and the consequent exhaustion obliged him to keep his bed. About the first of December he began to pass in his stools small masses of what appeared to be portions of intestine. Two to five pieces were noticed each twenty-four hours for four or five days; after which no more were seen. They varied in size from mere threads to pieces one half to one inch wide by an inch and a half long; and were irregular in shape. One other symptom was developed about the middle of January. After a severe paroxysm of pain lasting for several hours two to three ounces of blood passed his bowels; otherwise the symptoms continued unchanged, excepting the pain and tenderness of the bowels and tumor were much increased. Death closed his sufferings by exhaustion on the 20th of January, 1878, after a protracted illness of *eleven months.* During the whole period of the disease, when the patient was in a paroxysm of pain, he would frequently feel a gurgling in the tumor, as if something was passing through it; after which he would be relieved and experience an interval of ease.

Eight hours after death, with the assistance of Drs. Morgan and Beals of Conewango, N. Y., I made a *post mortem* examination. The colon was found to be the seat of an *invagination of some eighteen inches in extent.* The cæcum was inverted into the colon, drawing after it the ileum and corresponding mesentery. The inverted cæcum was swollen, indurated and ulcerated, with indications of considerable sloughing or necrosis having taken place. The cæcum was the only part that was swelled and indurated; and it formed a tumor within the ascending and transverse colon an inch and a half in diameter, and three to four inches long. This tumefied mass was movable within the colon to the extent of fourteen inches. There were no adhesions except within the inverted cæcum; and the bowel could be very easily drawn out till the tumor was reached. The passage through the tumefied cæcum was sufficiently large to admit my finger. Below the limit of the intussusception the colon and rectum were contracted to dimensions not more than half an inch in diameter. There were no evidences of either general or local peritonitis. The natural relation of the colon was very much changed, the whole of it having been dragged to the lower part of the abdomen. A portion of the transverse colon was found within the pelvis.

Several prominent physicians saw the above case, but no two could agree as to its nature. One regarded it as "enlargement of a mesenteric gland;" another as an "omental tumor;" another as "hypertrophy of the omentum;" another as "thickening and induration of the intestinal walls;" another as "*obstruction and enlargement of the pancreatic duct and gland;*" while others had their own peculiar version of the symptoms. As for myself, I did not become positive in my diagnosis till the latter part of the disease. Early in the case I hesitatingly suggested that it might be intussusception. Later, I unhesitatingly declared that it *was* intussusception. I was alone, however, in the diagnosis.

Being in doubt, at first, as to the import of the symptoms, and also being somewhat misled by the opinions of others whose counsel I valued, the case was allowed to go on till it was too late to attempt reduction by any means. Undoubtedly, it was a case in which proper application of distention to the colon might have effected reduction at anytime within the first four or five weeks. But the golden opportunity for either mechanical or operative measures was lost, because of a doubtful diagnosis in the earlier stage of the disease.

The question arises, "How could a positive diagnosis have been determined?" To this, I see but one answer, viz.: *By an exploratory incision.* But would it be justifiable in such cases? The answer to this depends upon the danger attendant upon such incisions, and explorations of the abdominal cavity. *What is the danger?* Who will answer?

